# Influence of device configuration and noise on a machine learning predictor for the selection of nanoparticle small-angle X-ray scattering models

**DOI:** 10.1107/S2053273324007988

**Published:** 2024-09-23

**Authors:** Nicolas Monge, Massih-Reza Amini, Alexis Deschamps

**Affiliations:** a Xenocs, Grenoble, France; b LIG, University of Grenoble Alpes, CNRS, Grenoble, France; c SIMaP, University of Grenoble Alpes, CNRS, Grenoble INP, Grenoble, France; Deutsches Electronen-Synchrotron, Germany

**Keywords:** SAXS, small-angle X-ray scattering, nanoparticles, machine learning, noise, configuration

## Abstract

Small-angle X-ray scattering (SAXS) is a widely used method for nanoparticle characterization. A common approach to analysing nanoparticles in solution by SAXS involves fitting the curve using a parametric model that relates real-space parameters, such as nanoparticle size and electron density, to intensity values in reciprocal space. Selecting the optimal model is a crucial step in terms of analysis quality and can be time-consuming and complex. Several studies have proposed effective methods, based on machine learning, to automate the model selection step. Deploying these methods in software intended for both researchers and industry raises several issues. The diversity of SAXS instrumentation requires assessment of the robustness of these methods on data from various machine configurations, involving significant variations in the *q*-space ranges and highly variable signal-to-noise ratios (SNR) from one data set to another. In the case of laboratory instrumentation, data acquisition can be time-consuming and there is no universal criterion for defining an optimal acquisition time. This paper presents an approach that revisits the nanoparticle model selection method proposed by Monge *et al.* [*Acta Cryst.* (2024), A**80**, 202–212], evaluating and enhancing its robustness on data from device configurations not seen during training, by expanding the data set used for training. The influence of SNR on predictor robustness is then assessed, improved, and used to propose a stopping criterion for optimizing the trade-off between exposure time and data quality.

## Abstract 2

This paper presents an enhanced model selection method for nanoparticle characterization via SAXS, improving prediction robustness across diverse instrumental configurations and varying noise levels, while optimizing data acquisition time on laboratory instrumentation.

## 1. Introduction

Small-angle X-ray scattering (SAXS) is a characterization technique widely used in the field of material science (Saurel *et al.*, 2019 ▸; Jouault *et al.*, 2010 ▸) and biology (Lombardo *et al.*, 2020 ▸; Dyer *et al.*, 2014 ▸; Kirby & Cowieson, 2014 ▸) to analyse the shape (Wang *et al.*, 2010 ▸) and size distribution (Rattanawongwiboon *et al.*, 2022 ▸) of nanoparticles or biological molecules such as proteins (Kikhney & Svergun, 2015 ▸; Simpson *et al.*, 2020 ▸). Following data acquisition, the classical approach to analysing SAXS data involves several steps. The first step is data reduction: the elimination of cosmic X-rays from the signal, background noise subtraction, azimuthal integration to reduce 2D data to 1D data when the particle distribution is isotropic, and intensity normalization (Hopkins *et al.*, 2017 ▸). Once the data have been pre-processed, the first step of the particle analysis is to select the appropriate nanoparticle model to fit the SAXS curve, in the case where the particle shape and size distribution are not known beforehand. The scientific community has developed more than 200 models, such as those listed in the software *SasView* (https://www.sasview.org/), 70 of which are associated directly with nanoparticle shapes. The analyst usually selects several models likely to correspond to the sample, based on *a priori* knowledge. Once the models have been pre-selected, their parameters need to be initialized based on the likely range known from material processing, and the analyst performs a fit of the experimental curve using optimization algorithms such as the Levenberg–Marquardt (Moré, 1978 ▸) or *DREAM* (Vrugt *et al.*, 2009 ▸); finally, the analyst retains the optimized model that best fits the data.

This method can give rise to a number of difficulties: firstly, optimization algorithms are very sensitive to model initialization; therefore, it can be tedious to find a good parameter initialization for several models, particularly for models where the number of parameters is high, with no guarantee of avoiding convergence towards local minima. It is therefore crucial to use a very restricted pre-selection of models to avoid carrying out a time-consuming fit for a model that is ill-suited to the experimental data. To speed up and simplify the nanoparticle model selection phase, several studies have proposed using a data-driven approach to train machine learning predictors to automatically select the nanoparticle model that is most likely to be optimal. These approaches are facilitated by the possibility of rapidly simulating a large number of SAXS curves associated with different nanoparticle models to build up data sets used to train and test the predictors (Archibald *et al.*, 2020 ▸; Franke *et al.*, 2018 ▸; Tomaszewski *et al.*, 2021 ▸; Yildirim *et al.*, 2024 ▸). More recently, it has been shown that training deep convolutional neural network (CNN) predictors on a database of simulated SAXS curves enables accurate association of a nanoparticle shape to a simulated input (Monge *et al.*, 2024 ▸; Yildirim *et al.*, 2024 ▸). Our study also showed that the classification rules learned by the predictor were applicable to real SAXS curves; however, the results on real data deteriorate significantly when patterns have characteristics that do not exist in the training database. Since it has been verified that the sample characteristics (electronic contrast, volume fraction, dispersity) were included in the training data distribution, these differences between trained and tested patterns may be due in part to differences between the configuration of the instrument simulated in the training database and that actually used to acquire the real data, to differences in noise levels, or to other experimental complexity (polydispersity function, additional sources of scattering).

SAXS instrumentation comes in a wide variety of configurations and parameters. High-brilliance sources are available at synchrotron facilities, enabling very low noise data to be obtained with small acquisition times, while laboratory devices have less powerful sources with wider beams that generate a smearing effect, and lower photon flux resulting in lower signal-to-noise ratio (SNR) in the data. Most devices have sample-to-detector distances that can be set over a wide range, variable photon energy, and there are many types of photon detectors with varying numbers and sizes of pixels, which creates great variability in the ranges of scattering vector *q* obtained over different experiments. A universal predictor must necessarily be robust to variations in the *q*-vector range. The first objective of the present report is to assess the robustness of the predictor proposed by Monge *et al.* (2024 ▸) to variations over *q*-space ranges, and to show that a single predictor trained on several different *q* ranges is robust to configurations that were not seen during the training step. Robustness to different noise levels is another essential criterion for a universal predictor, all the more important as the use of laboratory SAXS instruments for the analysis of low-contrast samples strongly penalizes the exposure time versus SNR trade-off. Proposing a predictor robust against low-SNR input data and determining the minimum SNR necessary for a good prediction can help determine the optimal measurement time for obtaining reliable results. The second objective of this study is therefore to evaluate the performance of the predictor proposed by Monge *et al.* (2024 ▸) as a function of the SNR level.

## 2. Proposed approach

### 2.1. Influence of device configuration

SAXS instrumentation presents a wide variety of devices and great flexibility in their settings. The sample-to-detector distance and detector characteristics vary the range of *q* space over which the curve is represented. Equivalently, variations of photon energy also change this range of scattering vectors. A nanoparticle model predictor must be robust to variations in *q*-space ranges, and must be able to make accurate predictions on data represented over *q*-space ranges that have never been seen during the training phase. To assess the robustness of the predictor described by Monge *et al.* (2024 ▸), a database composed of SAXS curves simulated from nine nanoparticle models was generated. This data set is the same as the one described by Monge *et al.* (2024 ▸), which contains 4184 *I*(*q*) curves for each of the following nanoparticle models: sphere, oblate ellipsoid, prolate ellipsoid, cylinder, core–shell sphere, core–shell prolate, core–shell oblate, core cylinder and hollow sphere. These particle form factors were chosen as being illustrative of classical nanoparticle shapes, but the methodology presented here is naturally applicable to other form factors. For each form factor the different parameters were varied randomly (dispersity, contrast, shape parameters). In the following, this data set is split between train and test in the proportion of 90%/10%. Several device configurations are applied to this theoretical data set to generate nine realistic data sets which take into account the experimental parameters of a device. The nine device configurations used represent a Xenocs Xeuss 3.0 device in various configurations. Some parameters are common to all configurations: the detector is a Dectris Eiger1M, the source is Cu with a wavelength of 1.54 Å and intensity of 9.27 × 10^6^ photons s^−1^, and exposure time is 20 min. The configurations differ in sample-to-detector distance 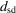, which also results in a variation in beam width represented by the FWHM value, expressed in Å^−1^. The values of FWHM are chosen empirically from measurements on real devices. The sample-to-detector distances are between 900 and 4500 mm, and scaled to obtain a linear variation between the 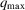 values. Table 1 ▸ details the *q*-space ranges associated with the sample-to-detector distances.

The predictor described by Monge *et al.* (2024 ▸) is composed of a set of pre-processing steps, a representation transformer of CNN type and a classifier of the type XGBoost (eXtreme Gradient Boosting) (Chen & Guestrin, 2016 ▸); it was implemented using *Tensorflow* (v.2.5.0) (Abadi *et al.*, 2016 ▸) and *XGBoost* (v.1.5.2) and trained on an Nvidia RTX 3080 GPU. Details of the architecture and hyperparameters used are available in Appendix *A*[App appa]. To evaluate the generalizability of the predictor to several configurations, it is first trained in a mono-configuration scenario, then in a multi-configuration scenario. In mono-configuration training, a specific predictor is trained on a data set associated with a single device configuration, then tested on a data set associated with this same configuration, for all configurations. Multi-configuration training consists of training a predictor on more than one configuration. In the all-configurations scenario, the predictor is trained and tested on the nine configurations. During the training step, it is therefore exposed to the same samples simulated in nine different device configurations. Other multi-configuration training scenarios are explored to test the robustness of the predictor to data acquired via a new device configuration: unseen-configuration training consists of training a predictor on some device configurations and testing it on data simulated with configurations absent from the training data set.

### 2.2. Noise influence

While it is possible to obtain data with a high SNR in less than a second in synchrotron facilities, laboratory instrumentation with less powerful sources implies that a trade-off has to be made between sample exposure time, which can last several tens of minutes, and the SNR of the acquired data. This trade-off is all the more critical when the sample to be analysed has a low electronic contrast between the particles and the solvent, or when the sample has a low concentration. On the other hand, optimizing this trade-off is important when the experiment requires the user to monitor *in situ* reactions whose kinetics are close to those of the exposure time. In this scenario, establishing a criterion based on the SNR to enable the user to know when an acquisition can be completed, and proposing analysis tools efficient even in a low-SNR context are two ways of improving the exposure time versus SNR trade-off.

Four training data sets are generated by associating the simulated data set mentioned in Section 2.1 with several device configurations. Each training data set is simulated with a different exposure time: 1, 30, 1200 and 2000 s. The fixed parameters are as follows: the detector is a Dectris Eiger1M, the source is Cu with a wavelength of 1.54 Å and an intensity of 9.27 × 10^6^ photons s^−1^ . Unlike in Section 2.1 in which the data in a data set consisted of a single configuration, in this section a training data set contains the data simulated from all the (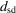, FWHM) pairs described in Section 2.1. The SNR of a SAXS curve is influenced not only by exposure time but also by factors such as sample concentration and electronic contrast, or background correction. Consequently, the data set encompasses a spectrum of SNR values, with this spectrum fluctuating in relation to exposure time. Ten test data sets were also generated, associated with the following exposure times: 1, 30, 110, 240, 410, 630, 890, 1200, 1600 and 2000 s, allowing the predictor to be evaluated over a wide range of SNR. In order to reduce the computation time allocated to the simulations, these test data sets are composed solely of simulated data with a sample-to-detector distance of 1800 mm and with an FWHM of 0.00165 Å^−1^. As in Section 2.1, various training scenarios are explored. In mono-time training, the predictor is trained on one of the training data sets and tested on all test data sets. In multi-time training, the predictor is trained on the entirety of the training data sets and subsequently tested on the complete set of test data sets.

In the simulated SAXS curves, the noise level is influenced by the intensity detected by the device and follows a Poisson distribution. This means that the SNR changes depending on the intensity value along the curve, which varies by several orders of magnitude. If we neglect the noise arising from the buffer subtraction, at a given *q* value the SNR is defined as 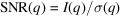, 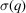 being the standard deviation of the noise on the SAXS curve. In practice, 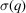 can be estimated easily during azimuthal integration under the two following assumptions: only Poisson noise is present and detector pixels are independent: 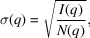
where 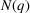 is the number of 2D image pixels used to compute the 1D intensity value 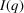 at the azimuthal integration step. See Appendix *B*[App appb] for the proof.


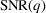
 can then be expressed as follows: 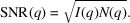
For simplicity of analysis we need a single SNR value per curve. Therefore, we have chosen to define the SNR of a curve as the SNR computed at the value of *q* where the contribution to integrated intensity 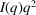 is maximal. The SNR values will therefore be large ones, not representative of parts of the curve where the data are noisy, but this criterion makes it possible to compare samples with different particle size or electronic contrast.

## 3. Results and discussion

### 3.1. Multi-configuration training

Training the predictor under various training scenarios involves distinct steps. In the mono-configuration training scenario, for each device configuration, a predictor is trained on the associated training data set specific to that configuration and subsequently tested on the test data set corresponding to the device configuration. In the multi-configuration training scenario, a predictor is trained across all device configurations and tested against the complete set of test data sets. The third training scenario, termed ‘unseen-configurations training’, is further divided into sub-scenarios. The core idea of this experiment is to evaluate the predictor’s performance on device configurations it has not encountered during training. In the 8-seen-configurations scenario, the predictor is trained on eight out of the nine configurations (configs) and tested on the configuration absent from its training. This experiment is executed across all test data sets. Within the 5-seen-configs, 3-seen-configs and 2-seen-configs scenarios, training is conducted across multiple configurations, and the predictor’s performance is assessed on configurations 1000, 1290, 1500 and 2250 mm, which were not seen during training. The data sets utilized for training are as follows:

5-seen-configs: 900, 1130, 1800, 3000, 4500 mm.

3-seen-configs: 900, 1130, 4500 mm.

2-seen-configs: 900, 4500 mm.

**Figure 1:**
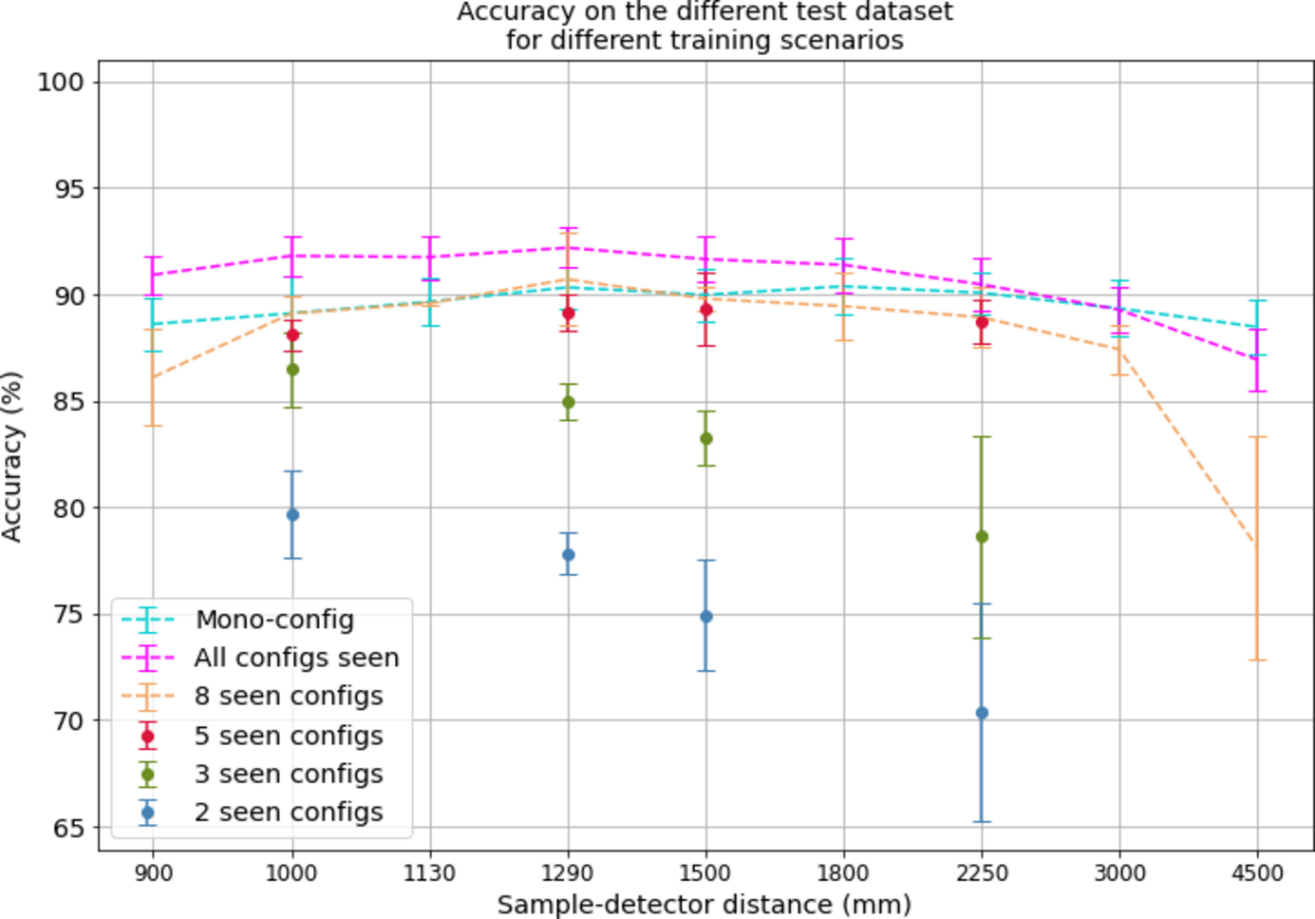
Mean accuracy of the predictor over training sessions for each test data set in the different training scenarios. Uncertainty corresponds to 3σ, with σ the standard deviation of the accuracy over training sessions. The *x* axis corresponds to the sample-to-detector distance of the test data set configuration.

Fig. 1 ▸ presents the results obtained for the different training scenarios. Regardless of the scenario, predictors are trained and tested five times on the same data batches, and the results presented in Fig. 1 ▸ correspond to the mean accuracy over the five training sessions. The uncertainty in the results is represented by three times the standard deviation of the accuracy across the training sessions.

On most test data sets, the accuracy achieved with the all-configuration training scenario surpasses that obtained through mono-configuration training. Mono-configuration training outperforms all-configuration training only on the test data set associated with a distance of 4500 mm. Across device configurations ranging from 900 to 2250 mm, results are enhanced by 0.4 to 2.3 accuracy points by an all-configuration training, indicating a significant improvement. On the test data set associated with the 3000 mm configuration, both training scenarios yield comparable performances, while the all-configuration training results in a 1.6 accuracy point decline on the test data set associated with the 4500 mm configuration. Thus, all-configuration training offers a dual advantage over mono-configuration training. First, it enhances the predictor’s reliability in its classification task. Moreover, it makes model deployment easier by necessitating only a single predictor instead of one per device configuration. This not only reduces the software’s memory footprint but also eliminates the need to manage predictor selection based on the device configuration used for input data acquisition.

Let us now compare the results of the all-configuration scenario with those of the unseen-configuration scenarios. A decrease in accuracy is observed in the 8-seen-configurations scenario, *i.e.* when the predictor is trained on all configurations except the test one, compared with the all-configurations training. This decrease in accuracy ranges from 1.6 to 2.7 points for configurations associated with intermediate distances of 1000 to 3000 mm, and is more pronounced at extrema distances with a loss of 4.8 points at 900 mm and 8.8 points at 4500 mm. The training scenario of 5-seen-configurations remains at a lower yet acceptable accuracy; however, the 3-seen-configurations and 2-seen-configurations lead to even larger losses in accuracy as the number of configurations seen during training decreases.

When the predictor is trained using all configurations, it performs very well on configurations it has already seen. However, when we test it on a configuration not included in its training set, like in the 8-seen-configurations scenario, there is a noticeable but minor drop in accuracy. This drop is less significant when the test configuration is surrounded by similar configurations in the training data. Specifically, when we remove configurations similar to the test one from the training set, the drop in accuracy becomes more pronounced. In the 8-seen-configurations scenario, the predictor’s accuracy decreases more when tested on configurations with higher 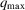 or lower 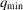 than those on which the predictor has been trained. This highlights that having a dense training set around unseen test configurations is crucial for accurate predictions. Therefore, training the predictor with a comprehensive range of device configurations ensures its reliability, especially when dealing with new configurations or *q*-space ranges.

### 3.2. Influence of noise level on prediction accuracy

**Figure 2:**
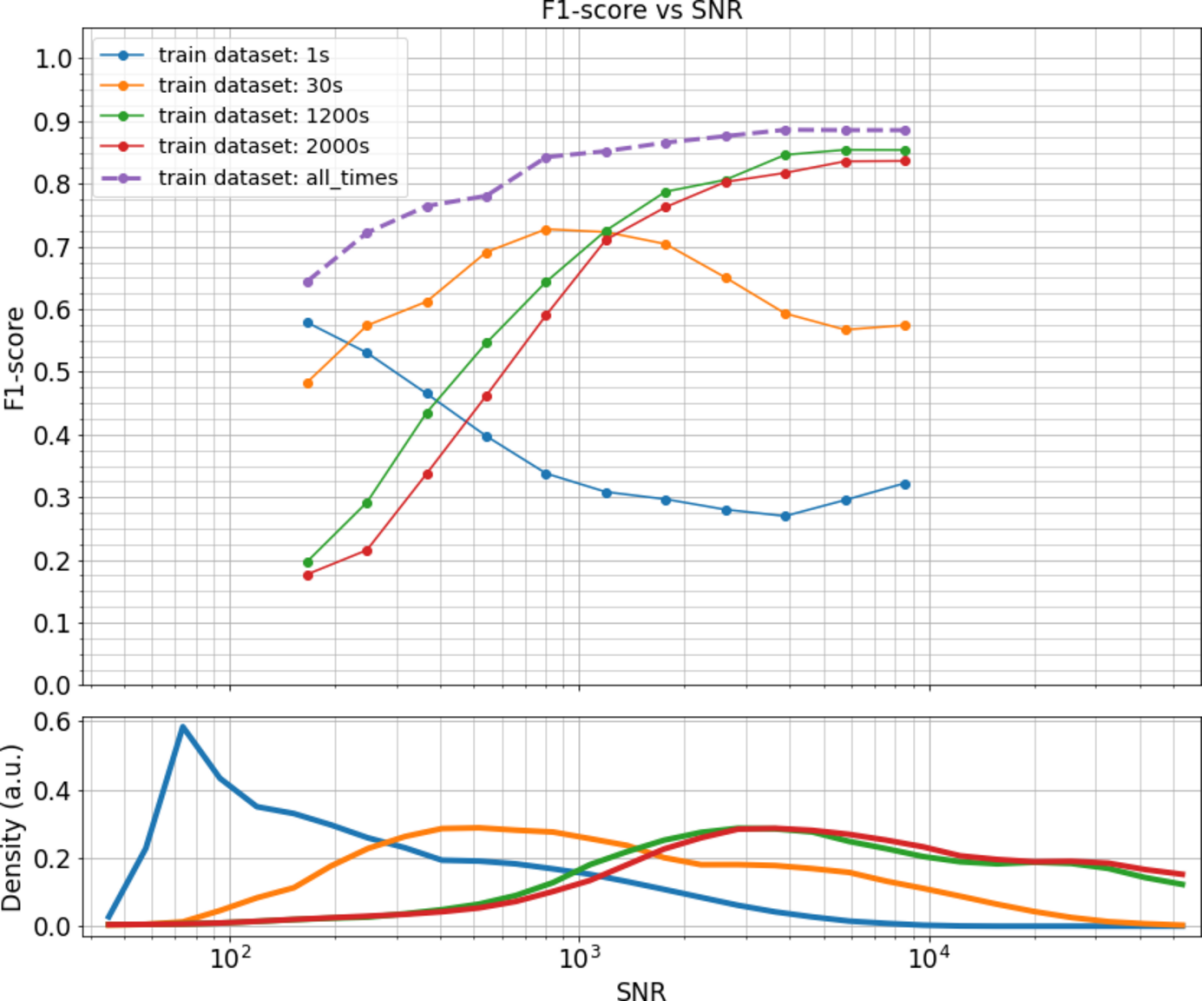
The upper graph represents the mean macro F1-score of the predictor for the different training scenarios, against the SNR of the tested data. The lower graph represents the SNR histograms of the different training data sets.

In this section, we compare various training scenarios to gauge how the predictor’s robustness evolves across different SNR. Specifically, we examine the SNR of the data to which the predictor is exposed during training. Fig. 2 ▸ provides the density of SNR levels in each of the training data sets and a comparative analysis of the predictor’s macro F1-score for each of the different scenarios. Macro F1-score is chosen instead of accuracy to take into account the imbalance of the labels at a given SNR.

Training the predictor on data sets with exposure times of 1200 and 2000 s proves effective, achieving an F1-score exceeding 0.8 for data with an SNR above 2500. However, as the SNR decreases, the performance diminishes sharply: at an SNR of 300, the F1-score drops to 0.4. Conversely, training on data sets with 1 or 30 s exposure times enhances the predictor’s resilience at low SNR. Yet, this approach leads to a notable decrease in performance at high-SNR levels, yielding results inferior to those obtained from the 1200 and 2000 s data sets when dealing with high-SNR data. Training on all four data sets concurrently offers a balanced approach. It captures the advantages of individual data set training without inheriting their respective limitations. Adopting this multi-dataset training strategy consistently yields superior accuracy rates across all SNR levels.

**Figure 3:**
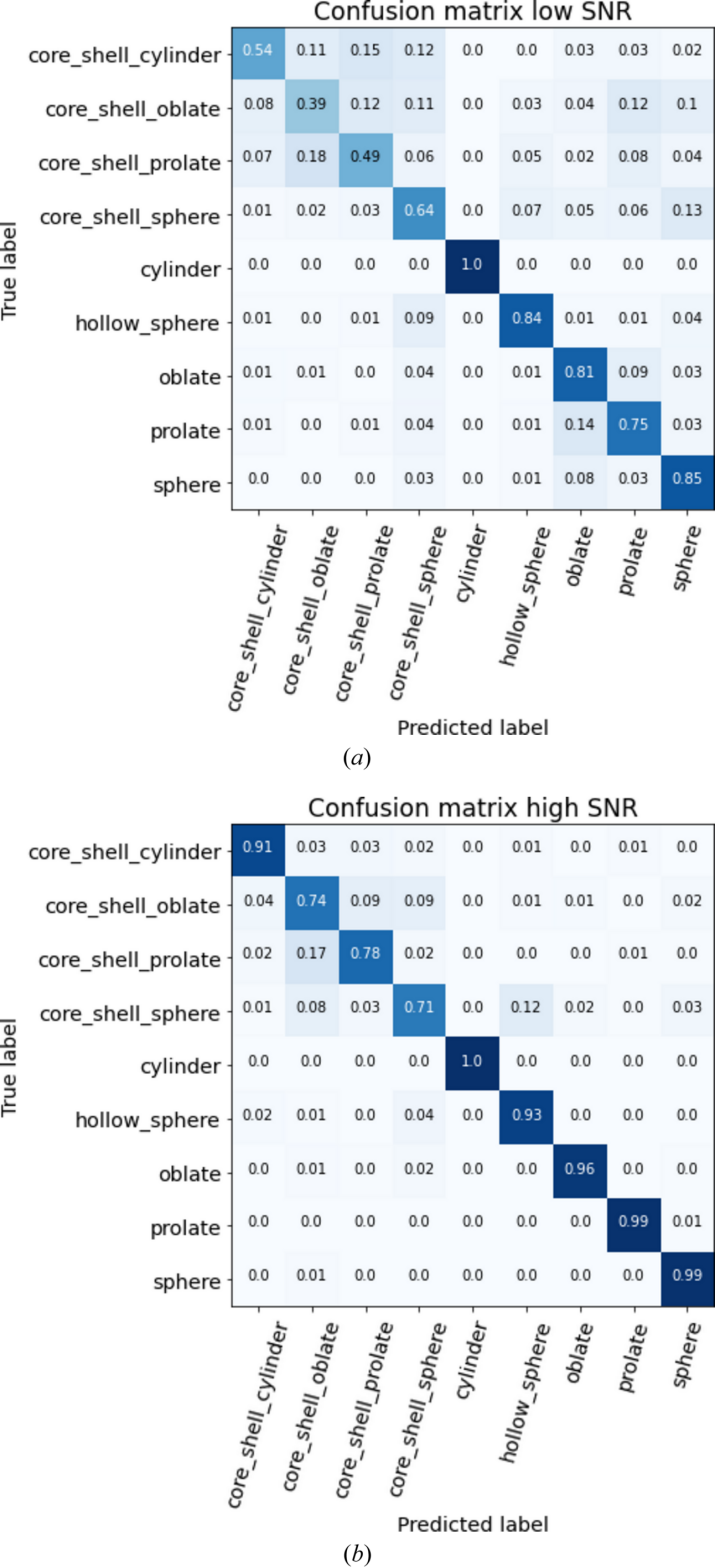
Confusion matrices of the predictor trained on the all-times data set. The confusion matrix for low SNR is derived from data with SNR between 100 and 400, while the high-SNR matrix uses data with SNR from 4000 to 9000. These confusion matrices are normalized by the number of predictions, so that the sum of a row is equal to 1. As an example, on (*a*) 85% of the spheres were well classified, 3% were confused with prolates, 8% with oblates, 1% with hollow spheres and 3% with core–shell spheres.

In the analysis presented in Fig. 3 ▸, the confusion matrices provide valuable insights into the efficiency of the predictor trained on the all-times scenario across various SNR levels. Specifically, in the high-SNR matrix, misclassifications predominantly occur among the core–shell models, which are mainly confused with each other. Among the 26% of misclassified core–shell oblates, the confusion is distributed as follows: 9% are confused with core–shell prolates, 9% with core–shell spheres, 4% with core–shell cylinders, 1% with hollow spheres, 1% with oblates and 2% with spheres. Notably, models with homogeneous electron densities, namely spheres, prolates, oblates and cylinders, exhibit minimal confusion. In contrast, the low-SNR matrix reveals a significant increase in misclassification rates for all models except cylinders. The predictor struggles to distinguish core–shell models, which are frequently misclassified among themselves and with models possessing homogeneous electron densities. This is likely related to the fact that the signal characteristic of the core–shell nature of the particle is mainly present at large values of the scattering vector where the signal noise is high. Actually, the predictor retains some efficacy with over 75% accuracy for sphere, prolate, oblate, cylinder and hollow sphere models. However, this represents a notable decline in performance compared with high-SNR conditions.

**Figure 4:**
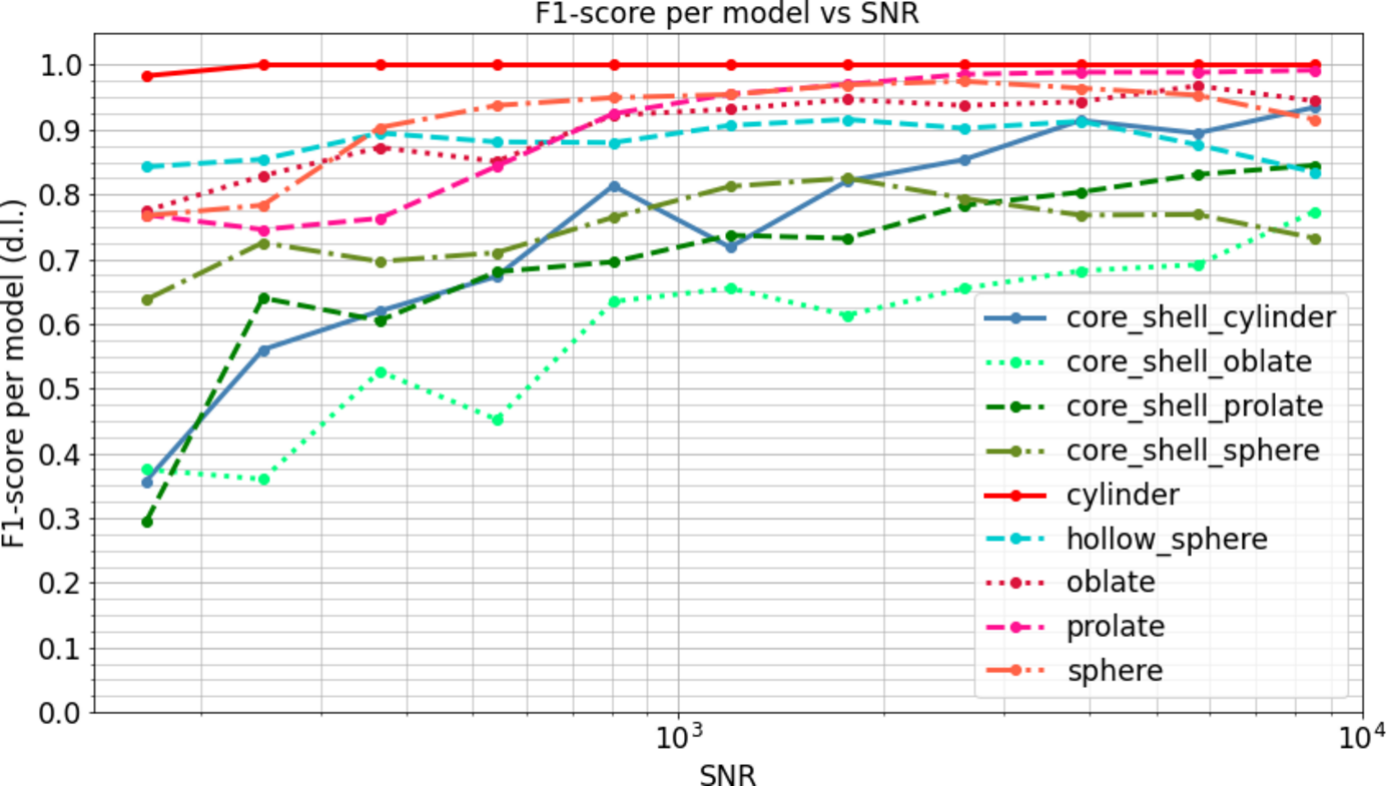
The graph represents the F1-score per model against the SNR of the tested data, obtained using the predictor trained on the all-times data set.

Fig. 4 ▸ provides a deeper insight into this analysis by displaying the F1-score per model across various SNR levels when the predictor is trained on the all-times data set. It is evident that starting from an SNR of 500, the F1-score surpasses 0.8 for all four homogeneous density models, which is close to the maximum value achievable by this model. In contrast, achieving an F1-score level of 0.7 requires an SNR above 800 for most of the non-homogeneous models and up to 6000 for core–shell oblates.

The predictor’s performance is influenced by the SNR of the input data. Exposing the predictor to a broad range of SNR values during the training phase enhances its efficacy across all SNR levels, with a notable improvement at lower SNR values. Understanding how the predictor’s capability to correctly match a SAXS curve to the appropriate model evolves with varying SNR, assuming the input data fall within the training distribution, allows for the establishment of minimum SNR thresholds for data acquisition. These thresholds vary depending on the specific use-cases: data with an SNR from 1000 to 10 000 appear necessary for analysing nanoparticle data potentially characterized by multiple electron densities. In contrast, an SNR greater than 500 should suffice for deriving insights from data originating from homogeneous nanoparticles. These absolute values are specific to the particular case of our study and may vary if different form factors, or a large number of them, were included in the database.

### 3.3. Outlook: validation on experimental data

Currently, no database containing labelled experimental SAXS curves is available to the scientific community, complicating validation efforts. Studies such as those of Archibald *et al.* (2020 ▸), Tomaszewski *et al.* (2021 ▸), Monge *et al.* (2024 ▸), Yildirim *et al.* (2024 ▸), which propose using machine learning approaches for the classification of SAXS data, have all been conducted using simulated data or very limited experimental data sets. While awaiting the opportunity to perform a statistical validation of the influence of device configuration or SNR on experimental data, we have evaluated the applicability of our approach to experimental data on a limited number of samples.

SAXS profiles of three samples of Au nanoparticles in solution were acquired on a Xenocs Xeuss 3.0 device with acquisition times varying from 1 to 20 min, resulting in an SNR between 180 and 1450. Samples were also characterized by transmission electron microscopy (TEM) after drying, using a Jeol 1010 instrument working at 100 kV located at IRAMIS/LIONS at CEA Saclay, allowing us to determine without ambiguity their form factor, as well as polydispersity, and to check that the aspect ratios of the nanoparticles are well within the limits defined during the simulations. One sample is labelled as spheres and two as prolate ellipsoids. An example of the SAXS profile is shown in Fig. 6.

**Figure 5:**
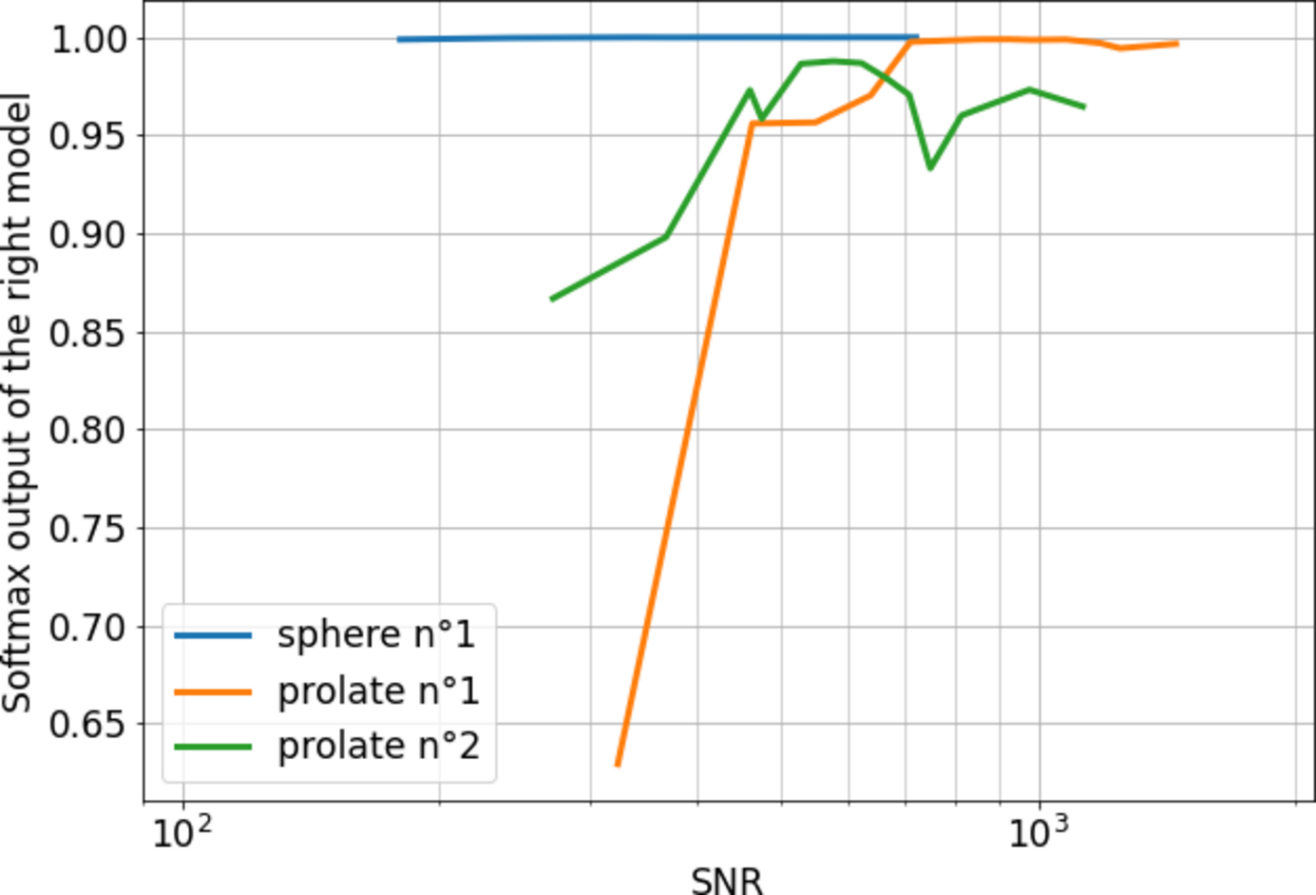
Evolution of the softmax output associated with the right model as a function of the SNR for three nanoparticle samples.

**Figure 6:**
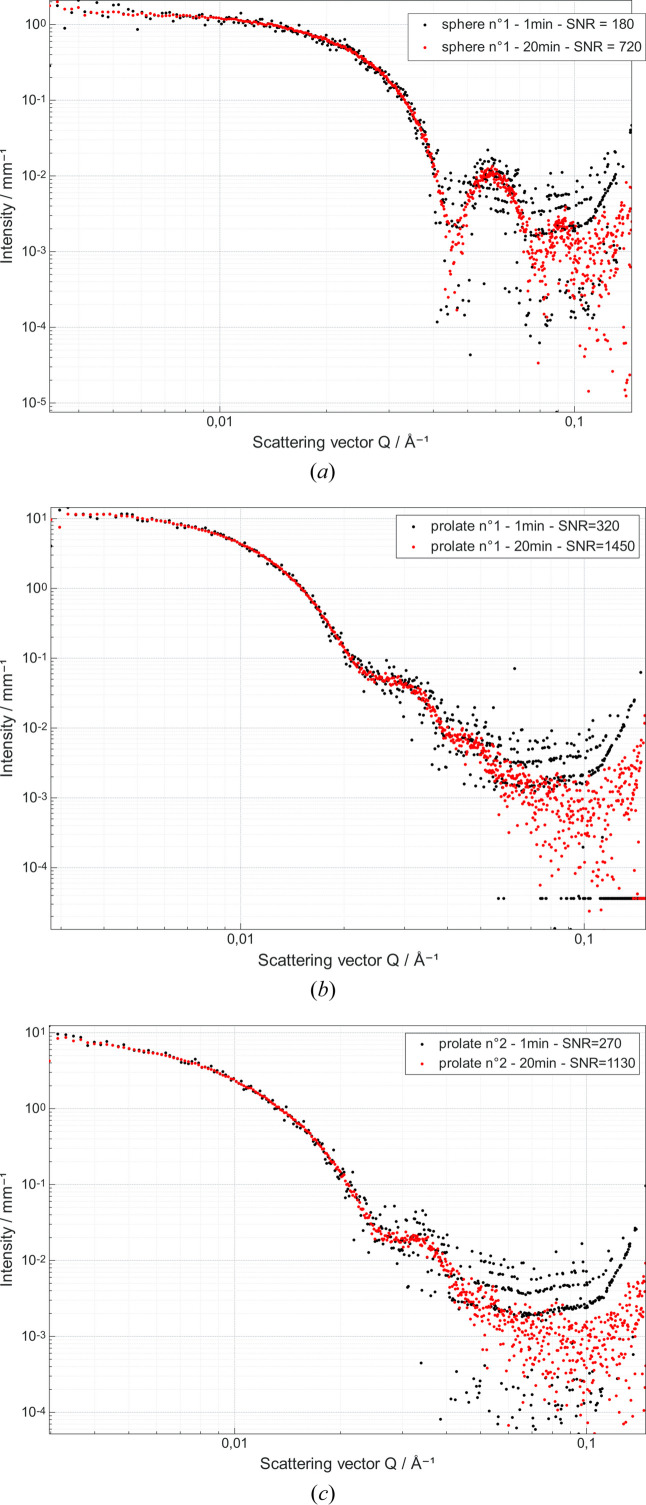
SAXS profiles of the three nanoparticle samples for acquisition times of 1 and 20 min. (*a*) Sphere no. 1, (*b*) prolate no. 1, (*c*) prolate no. 2.

Using the neural network classifier trained on simulated data presented above, in the all-SNR scenario, we observe in Fig. 5 ▸ the evolution of the softmax output corresponding to the correct model, as a function of the SNR. This measure, which is the most appropriate metric in the absence of a statistically representative data set, can be interpreted as a probability and represents the confidence of the predictor. However, this interpretation has its limitations, as the outputs of a neural network with softmax activation trained to minimize a cross-entropy loss are often subject to overconfidence (Pearce *et al.*, 2021 ▸).

For the sample of spherical nanoparticles, the softmax output remains close to 1 regardless of the SNR, indicating high classifier confidence even at a low SNR below 200. For the two prolate samples, the classifier’s confidence increases with the SNR, stabilizing at an SNR threshold of 700 for prolate sample 1 and around 400 for prolate sample 2. The better confidence of sphere prediction when compared with prolate is consistent with the shape-dependent accuracy determined on the simulated data. These examples demonstrate that the quality of predictions can be significantly affected by the SNR, and future access to a more comprehensive data set should enable precise validation of the SNR thresholds necessary for accurate predictions. This, in turn, would allow for the minimization of exposure time required for a sample.

## 4. Conclusion

In this paper, we have presented an investigation of the performance of a SAXS nanoparticle model predictor when subjected to data from various device configurations and different noise levels to assess its degree of robustness. The predictor was trained on different data sets containing data represented over one or multiple *q* ranges and tested on data represented over both seen and unseen *q* ranges during training. The predictor’s performance is enhanced by the presence of a variety of different *q* ranges in the training data, and it demonstrates excellent robustness to new device configurations when the training data are sufficiently similar to them, namely a comparable accuracy with that of configurations seen during training. Robustness to noise was evaluated by training the predictor on different data sets with varying SNR and testing it on data sets covering a broad range of SNR. The predictor exhibits significant performance variation: its performance is weak when the SNR is very low and above 0.8 in average when the SNR is very high. Performance across different SNR levels is influenced by the training data set, which must encompass a broad range of SNR to achieve optimal performance. This study makes it possible to identify minimum SNR thresholds that can be targeted during data acquisition to enable reliable use of the predictor, thus providing the experimenter with an objective criterion for determining the necessary exposure time for their experiment. One significant limitation of the classification approaches presented in this study and in other studies in the literature is the fact that it is currently limited to in-distribution data, namely the evaluated data should be well represented by the training database. In cases where a sample would be out of distribution (*e.g.* the form factor is not included in the training, sample with only noise *etc*.) the experimentalist may be deceived by the classification result. To solve this issue, we are currently working on the development and implementation of a specific model for out-of-distribution detection.

## Figures and Tables

**Table 1:** Sample-to-detector distances 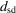 and beam FWHM used to simulate the various configurations and their associated *q*-space ranges

	SAXS configuration			
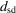 (mm)	FWHM (Å^−1^)	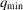 (Å^−1^)	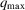 (Å^−1^)	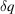 (Å^−1^)
900	0.00240	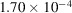	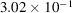	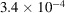
1000	0.00225	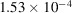	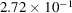	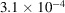
1130	0.00210	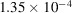	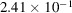	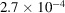
1290	0.00195	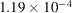	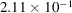	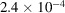
1500	0.00180	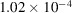	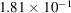	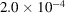
1800	0.00165	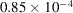	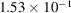	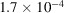
2250	0.00150	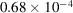	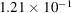	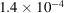
3000	0.00135	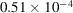	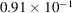	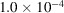
4500	0.00120	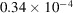	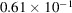	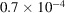

## Appendix A Predictor details

The predictor used throughout the study is the optimal predictor described by Monge *et al.* (2024 ▸). This consists of a CNN employed as a space transformer, and an XGBoost for the classification task. The network is trained using the Adam optimizer and categorical cross-entropy as loss function and its architecture is as follows:

1D convolutional layer [*n* filters: 64, kernel size: 7, activation function: ReLU (rectified linear unit)].

1D convolutional layer (*n* filters: 64, kernel size: 7, activation function: ReLU).

Max pooling operation (kernel size: 6).

Dropout operation (rate: 0.25).

1D convolutional layer (*n* filters: 64, kernel size: 7, activation function: ReLU).

1D convolutional layer (*n* filters: 256, kernel size: 7, activation function: ReLU).

Max pooling operation (kernel size: 6).

Dropout operation (rate: 0.25).

Global max pooling (output size: 256).

The XGBoost classifier is composed of 200 trees.

## Appendix B Noise estimation

Within the framework of probability theory and random signals, the intensity of the SAXS curve at a given *q* value is a random variable  of variance .  is the result of the azimuthal integration of the 2D image:

where  is the set of 2D image pixels used to compute the 1D intensity at the given *q* value, *N* the cardinality of  and  the random variable representing the intensity (*i.e.* the number of photons) received by a pixel *p*. We assume that all the  follow the same Poisson law of expectation *E*.

We then have

We assume that all pixels of the detector are independent two by two, so

As all the  follow a Poisson law of expectation *E*, we have

So, combining (2) and (3):

Considering that the user has access to one observation per random variable  (*i.e.* the user has access to one experimental image) and thus one observation of , we note those observations  and *I*, respectively, and we have

Expectation of the Poisson law can then be approximated by the average of the observations , and using (6) we have

and

The SNR of the 1D intensity at a given *q* value can then be computed from the observed image using this approximation:

## Appendix C Experimental data

Fig. 6 ▸ shows the SAXS profiles of the three Au nanoparticle experimental samples.
